# Splenic Vein Diameter is a Risk Factor for the Portal Venous System Thrombosis After Partial Splenic Artery Embolization

**DOI:** 10.1007/s00270-020-02751-8

**Published:** 2021-01-20

**Authors:** Satoyuki Ogawa, Akira Yamamoto, Atsushi Jogo, Mariko M. Nakano, Ken Kageyama, Etsuji Sohgawa, Norifumi Nishida, Toshio Kaminou, Yukio Miki

**Affiliations:** 1 Department of Diagnostic and Interventional Radiology, Osaka City University Graduate School of Medicine, 1-4-3 Asahi-machi, Abeno-ku, Osaka, Japan; 2 Department of Radiology, National Hospital Organization Osaka Minami Medical Center, Osaka, Japan; 3 Department of Diagnostic Radiology, Osaka Saiseikai Nakatsu Hospital, Osaka, Japan; 4 Department of Radiology, Tsukazaki Hospital, Himeji, Hyogo Japan

**Keywords:** Partial splenic artery embolization, Portal vein thrombosis, Portal venous system thrombosis, Maximum diameter of the splenic vein, Infarcted splenic percentage

## Abstract

**Purpose:**

Portal venous system thrombosis is a complication of partial splenic artery embolization, and pre-treatment risk assessment is thus important. The purpose of this study was to identify the risk factors for portal venous system thrombosis after partial splenic artery embolization.

**Materials and methods:**

We retrospectively analyzed 67 consecutive patients who underwent contrast-enhanced computed tomography before and after first partial splenic artery embolization between July 2007 and October 2018. As risk factors, we investigated age, sex, hematological data, liver function, steroid use, heparin use, and findings from pre- and post-treatment computed tomography. Uni- and multivariate analyses were performed to evaluate the relationship between thrombus appearance or growth and these factors. Values of *p* < 0.05 were considered significant.

**Results:**

Partial splenic artery embolization was technically successful in all 67 patients. Nine patients showed appearance or growth of thrombus. Univariate analysis showed maximum diameter of the splenic vein before treatment (*p* = 0.0076), percentage of infarcted spleen (*p* = 0.017), and volume of infarcted spleen (*p* = 0.022) as significant risk factors. Multivariate analysis showed significant differences in maximum diameter of the splenic vein before treatment (*p* = 0.041) and percentage of infarcted spleen (*p* = 0.023). According to receiver operating characteristic analysis, cutoffs for maximum diameter of the splenic vein and percentage of infarcted spleen for distinguishing the appearance or growth of thrombus were 17 mm and 58.2%.

**Conclusion:**

Large maximum diameter of the splenic vein before partial splenic artery embolization and high percentage of infarcted spleen after partial splenic artery embolization were identified as risk factors for portal venous system thrombosis.

**Level of Evidence:**

Level 4, Case Series

## Introduction

Partial splenic artery embolization (PSE) is widely used as a treatment for splenic injury, pancytopenia, ascites, esophagogastric varices, and portal hypertensive gastric disease. However, this procedure is also known to cause complications such as portal venous system thrombosis (PVST), splenic abscess, ascites, fever, and pain [1–8]. PVST refers to thrombus in the portal, splenic, and superior mesenteric veins. In particular, PVST is associated with an increased risk of death [9–11], and assessment of the risk of complications before PSE is thus important.

Hayashi et al. and Cai et al. reported an association between infarcted splenic volume and Child–Pugh score as risk factors for serious complications after PSE [1, 2]. Matsumoto et al. reviewed 16 cases of PSE and reported infarcted splenic volume as a significant risk factor for thrombus in the portal or splenic veins [3].

To the best of our knowledge, no reports have described the diameter of the splenic vein as a risk factor for PVST after PSE. Haag et al. have reported an association between splenic vein diameter and portal hypertension [12]. Measurement of splenic vein diameter on pre-treatment computed tomography (CT) is simple and easy. If we could confirm an association between diameter of the splenic vein before PSE and the appearance or growth of PVST after PSE, pre-treatment risk assessment would be easier. The purpose of this study was thus to investigate risk factors for the development of PVST after PSE, along with factors including diameter of the splenic vein.

## Materials and Methods

### Patients

This retrospective clinical study was approved by the ethics committee at our institution and was exempted from the requirement to obtain informed consent.

**Fig. 1:**
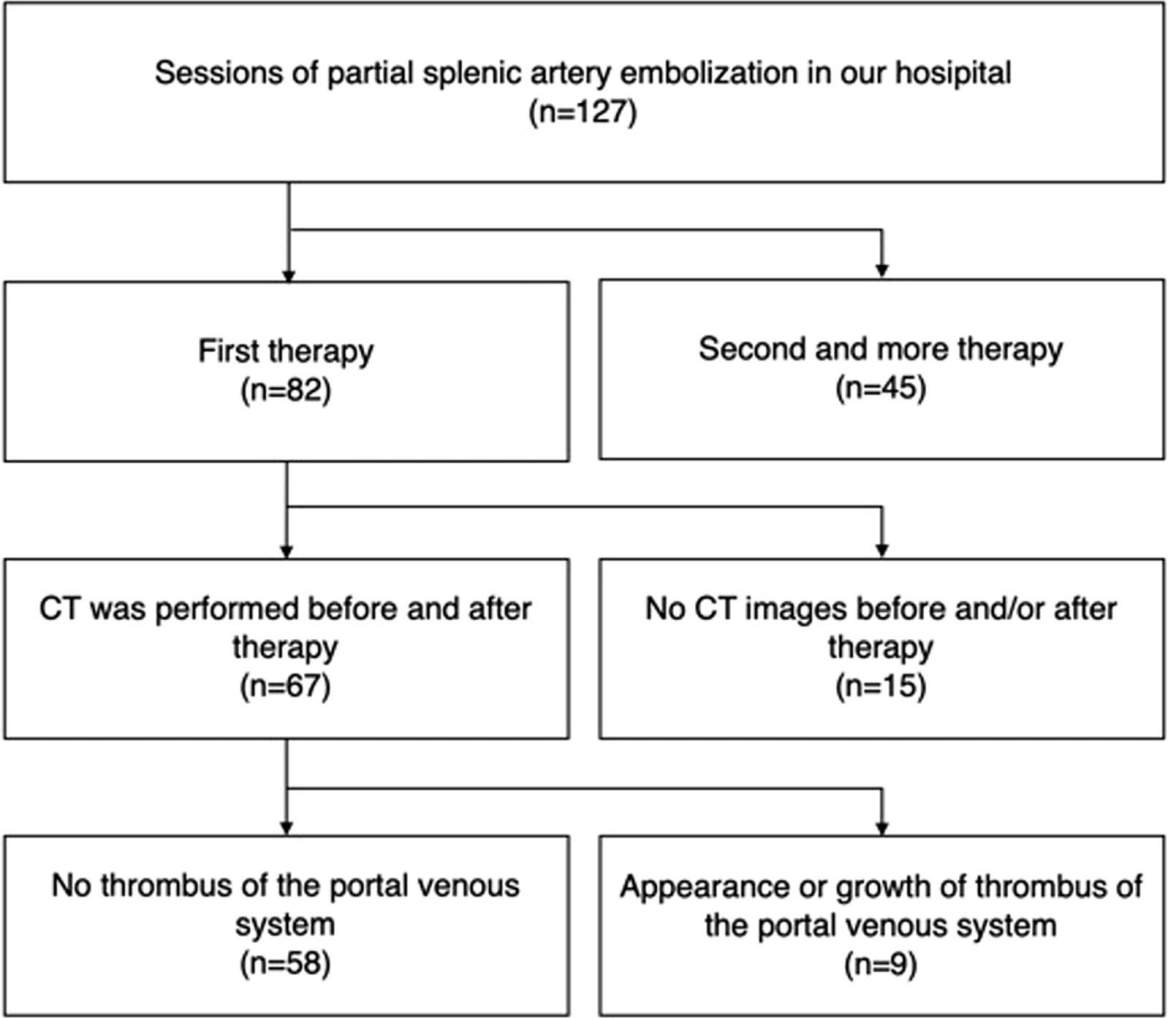
Flowchart for the study

Between July 2007 and October 2018, a total of 127 consecutive cases (76 patients in total) were treated with PSE at our institution. Forty-five cases that were receiving second or subsequent PSE were excluded. Of the remaining 85 cases, 67 cases (67 patients in total) underwent CT before and after PSE (Fig. 1).

### PSE Procedures

Under local anesthesia, a 4.0-Fr sheath was inserted into the common femoral artery. The celiac artery was selected using a 4.0-Fr catheter (RC-O9 or SHC; Medikit, Tokyo, Japan). A 1.7- to 2.2-Fr microcatheter was coaxially advanced into the splenic artery, and the tip of the catheter was inserted as distally as possible into a branch of the intra-splenic artery and embolized. As embolic materials, 1 to 2 mm cubes of gelatin sponge (Serescue®; Astellas, Tokyo, Japan or Spongel®; LTL Pharma, Tokyo, Japan), coils, Amplatzer vascular plug (Abbott Vascular, Santa Clara, CA), and N-butyl-2-cyanoacrylate (NBCA) (Histoacryl®; B Braun, Melsungen, Germany) were used alone or in combination. The embolic material used was gelatin sponge and coils in 28 cases (41.8%), coils in 18 cases (26.9%), gelatin sponge in 16 cases (23.9%), coils and NBCA in 2 cases (3.0%), NBCA in 1 case (1.5%), coils, NBCA, and gelatin sponge in 1 case (1.5%), and coils, plug, and gelatin sponge in 1 case (1.5%). Twenty-five cases received intra-arterial methylprednisolone sodium succinate (median, 125 mg; range, 50–500 mg) during treatment for the purpose of pain management. Cefazolin sodium was administered intravenously at 2 g/day for 4 days in all cases, for the purpose of preventing infectious complications. To prevent PVST, continuous infusion of heparin at 10,000 units/day was performed for 3 days from the date of treatment in 10 cases excluding emergency hemostasis cases after September 2015. Technical success was defined as the completion of embolization in one or more branches of the splenic artery.

### Evaluation Items

Before treatment, the following data were obtained for each patient: platelet count, prothrombin time, serum albumin, and alanine aminotransferase (ALT). All patients were evaluated for Child–Pugh classification and albumin-bilirubin (ALBI) grade. Contrast-enhanced CT (CECT) was used to determine the presence of hepatocellular carcinoma (HCC), ascites, PVST, and the volume and percentage of infarcted spleen. CT was performed using a 64-row multidetector-row CT system (Somatom Sensation 64; Siemens Medical Solutions, Erlangen, Germany) before and after PSE. Axial images were reconstructed in 5 mm slices in the arterial, portal, and delayed phases, respectively.

Delayed-phase images before PSE were used to determine total spleen volume, maximum diameter of the splenic vein, and presence of HCC, ascites, and PVST. Delayed-phase images after PSE determined maximum diameter of the splenic vein, non-infarcted splenic volume, infarcted splenic volume, and the appearance or growth of PVST. Pre- and post-PSE CECT were performed at a median of 34 days (range, 0–201 days) before PSE and 6 days (range, 1–78 days) after PSE, respectively. Pre-PSE CT was performed for other medical reasons, not specifically to evaluate PVST. Post-PSE CT was performed in all cases to evaluate the volume and percentage of infarcted spleen, PVST, and other complications. We examined whether intra-arterial steroid administration during treatment and intravenous heparin treatment were related to thrombus formation.

### Measurement of Maximum Diameter of the Splenic Vein and Splenic Volume

In all cases, total splenic volume before PSE and non-infarcted and infarcted splenic volumes after PSE were measured using the SYNAPSE VINCENT analyzer (Fujifilm Medical Co., Tokyo, Japan). For measurement, delayed-phase images reconstructed at 5 mm intervals were used. After importing pre- and post-PSE images into the SYNAPSE VINCENT analyzer, regions of non-infarcted and infarcted spleen parenchyma were selected manually, with a region of interest (ROI) in each slice. The selected ROI was automatically calculated as previously reported [13, 14]. The percentage of infarcted spleen was calculated as (volume of infarcted spleen/volume of infarcted spleen + volume of non-infarcted spleen) × 100 (%). Since comparison of thrombus size by visual assessment is difficult, thrombus volume was measured and quantitatively assessed to identify thrombus enlargement. The appearance or growth of PVST was evaluated by comparing results from CECT before and after PSE. Thrombus volume was similarly measured using the SYNAPSE VINCENT analyzer. The growth of PVST was defined as an increase in the volume of thrombus > 2.0 mL. Cases with appearance or growth of PVST were stratified according to the classification of Tsamalaidze et al. [15], which divides PVST into four types as follows: Type 1, asymptomatic thrombosis limited to the splenic vein; Type 2, asymptomatic thrombosis limited to the intrahepatic portal vein; Type 3, asymptomatic multiple or diffuse thrombosis of the portal venous system; and Type 4, symptomatic isolated, multiple, or diffuse thrombosis of the portal venous system. Maximum diameter of the splenic vein was manually measured on axial images before and after PSE. These tasks were performed independently by two radiologists with 8 and 11 years of experience, and values were finally determined using the mean values of each.

### Statistical Analysis

Univariate analyses were performed using GraphPad Prism version 5.02 software (GraphPad Software, San Diego, CA, USA). Univariate analysis was performed using the unpaired t test for continuous variables and Fisher’s test for other variables. Univariate analyses were performed for the following covariates: age, sex, platelet level, prothrombin time, total bilirubin, albumin, ALT, hepatic encephalopathy, ascites, HCC, ALBI score, pre-PSE maximum diameter of the splenic vein and total splenic volume, post-PSE volume of infarcted spleen, percentage of infarcted spleen, thrombus or occlusion of the portal venous system, use of steroid, and use of heparin. The paired t test was performed for the maximum diameter of the splenic vein before and after PSE. Multivariate analysis was performed using JMP version 9.0.2 software (JMP, SAS Institute, Cary, NC). In multivariate logistic regression with stepwise variable selection, the covariates were the same as in univariate analyses. Values of *p* < 0.05 were considered statistically significant in all analyses. The receiver operating curve (ROC) was drawn using JMP version 9.0.2 software. Youden index (sensitivity + specificity—1) was used as the cutoff value for the ROC.

## Results

Characteristics of these 67 patients (46 males, 21 females) are summarized in Table 1. Mean age was 57 ± 15 years (range, 9–80 years). Causes of liver cirrhosis were hepatitis C virus in 18 cases (26.8%), alcohol intake in 10 cases (14.9%), hepatitis B virus in 8 cases (11.9%), non-alcoholic steatohepatitis in 7 cases (10.4%), autoimmune hepatitis in 2 cases (3.0%), hepatitis B virus and alcohol intake in 1 case (1.5%), Budd-Chiari syndrome in 1 case (1.5%), and unclassifiable or unknown in 6 cases (9.0%). Fourteen patients (20.9%) showed no liver cirrhosis. According to ALBI grade [16], 13 cases were grade 1, 49 cases were grade 2, and 5 cases were grade 3. According to the Child–Pugh classification, the 53 cases with liver cirrhosis were categorized as A class in 17 cases, B class in 33 cases, and C class in 3 cases. Purpose of PSE was thrombocytopenia improvement in 38 cases (56.7%), treatment or relapse prevention of gastrointestinal tract varices in 15 cases (22.4%), treatment for splenic injury in 7 cases (10.4%), thrombocytopenia improvement and treatment or relapse prevention of gastrointestinal tract varices in 5 cases (7.5%), preoperative blood flow change in 1 case (1.5%), treatment of ascites in 1 case (1.5%). Mean total spleen volume before PSE in all patients was 711.8 ± 473.2 mL (range, 27.4–2203.0 mL). PSE was technically successful in all 67 cases. Mean total splenic volume, mean infarcted splenic volume, and mean percentage of infarcted spleen after PSE were 811 ± 541 mL (range, 23.9–2385 mL), 348 ± 321 mL (range, 1.4–1261 mL), and 39.8 ± 20.3% (range, 2.7–84.2%), respectively (Table 1).

**Table 1:** Characteristics of the 67 patients

Characteristic	Data
Age, mean ± SD (range)	57 ± 15 (9–80)
Sex, male/female	46/21
*Etiology, n (%)*	
Hepatitis C virus	18 (26.8%)
Alcohol intake	10 (14.9%)
Hepatitis B virus	8 (11.9%)
Non-alcoholic steatohepatitis	7 (10.4%)
Autoimmune hepatitis	2 (3.0%)
Hepatitis B virus and alcohol intake	1 (1.5%)
Budd-Chiari syndrome	1 (1.5%)
Unclassifiable or unknown etiology	6 (9.0%)
No liver cirrhosis	14 (20.9%)
*Assessment of liver function*	
No cirrhosis/Child–Pugh A/Child–Pugh B/Child–Pugh C	14/17/33/3
ALBI score, mean ± SD (range)	− 2.12 ± 0.51 (− 3.19 – − 0.84)
ALBI grade, 1/2/3	13/49/5
*Purpose of PSE*	
Thrombocytopenia improvement	38 (56.7%)
Treatment or relapse prevention of GI tract varices	15 (22.4%)
Treatment for splenic injury	7 (10.4%)
Thrombocytopenia improvement and treatment or relapse prevention of GI tract varices	5 (7.5%)
Preoperative blood flow change	1 (1.5%)
Treatment of ascites	1 (1.5%)
*Laboratory tests before PSE*	
Platelet count (× 10^4^/L), mean ± SD (range)	8.3 ± 6.7 (1.3–28.3)
ALT (IU/L), mean ± SD (range)	30 ± 24 (5–150)
Albumin (g/dL), mean ± SD (range)	3.47 ± 0.51 (2.3–4.4)
Total bilirubin (mg/dL), mean ± SD (range)	1.24 ± 0.78 (0.3–3.9)
Prothrombin time (%), mean ± SD (range)	68.8 ± 20.3 (32–139)
*CT before PSE*	
Ascites, absence/mild/moderate to severe	45/15/7
HCC, absence/presence	63/4
Occlusion or thrombus of portal venous system, absence/presence	43/24
Maximum diameter of splenic vein (mm), mean ± SD (range)	12.3 ± 5.1 (4–28)
Total splenic volume (mL), mean ± SD (range)	711.8 ± 473.2 (27.4–2203.0)
*CT after PSE*	
Total splenic volume (mL), mean ± SD (range)	810.6 ± 541.0 (23.9–2385.4)
Infarcted splenic volume (mL), mean ± SD (range)	348.1 ± 320.6 (1.4–1261.1)
Percentage of infarcted spleen (%), mean ± SD (range)	39.8 ± 20.3 (2.7–84.2)
Portal venous system thrombosis, no change/appearance or development	58/9
Maximum diameter of splenic vein (mm), mean ± SD (range)	11.6 ± 5.0 (4–27)
Steroid used for pain management, not used/used	42/25
Heparin used after PSE, not used/used	57/10

*ALBI* albumin-bilirubin; *PSE* partial splenic artery embolization; *GI* gastrointestinal; *ALT* alanine aminotransferase; *CT* computed tomography

### Appearance or Growth of PVST After PSE

**Fig. 2:**
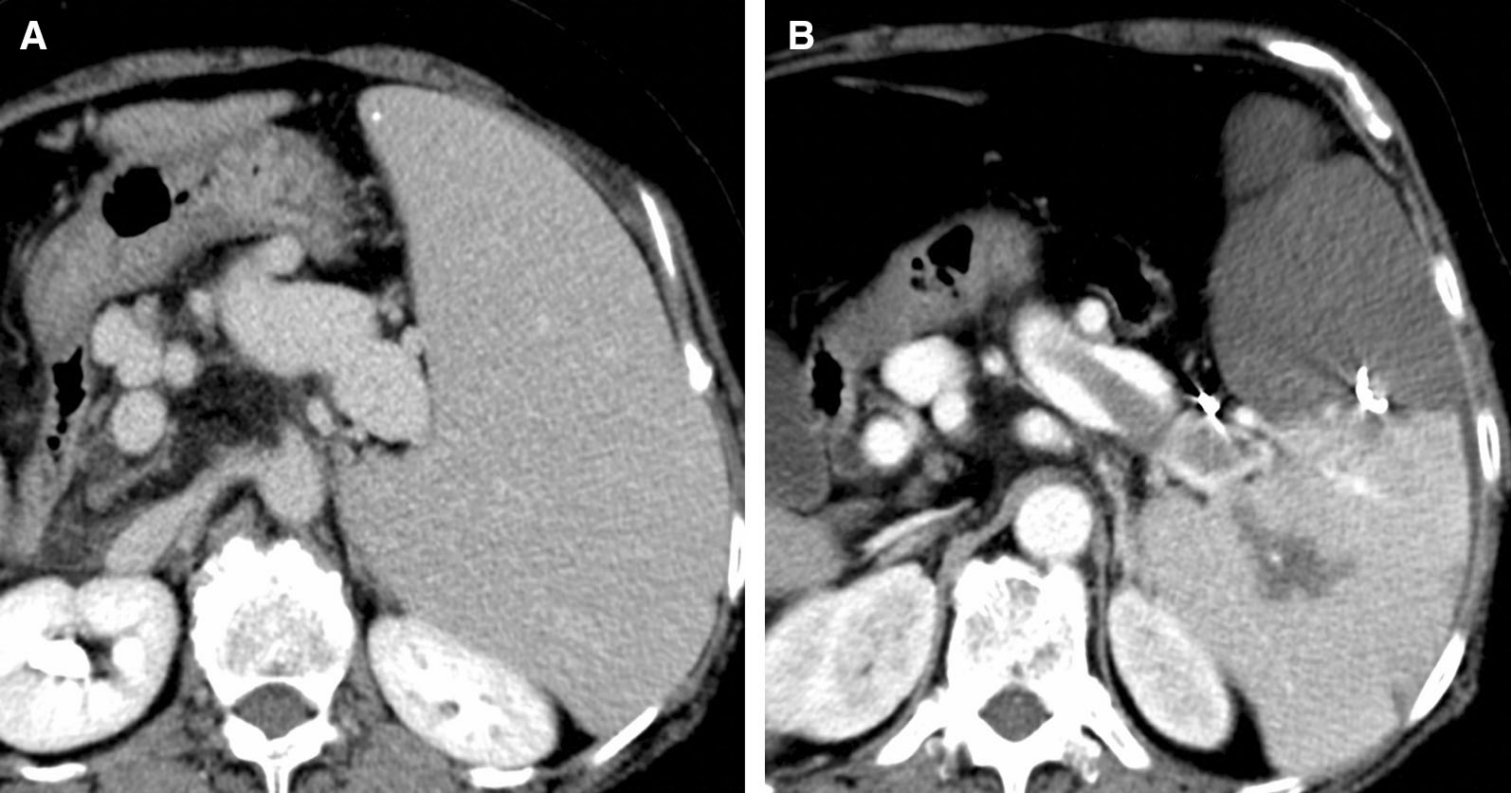
A 60-year-old woman underwent PSE for thrombocytopenia. **A** Abdominal CECT before PSE shows no thrombus in the splenic vein. **B** Abdominal CECT after PSE shows a large thrombus in the splenic vein. Infarcted splenic volume after PSE in this patient was 707.1 mL, and percentage of infarcted spleen was 60.1%

Nine of 67 cases (13.4%) showed appearance or development of PVST after PSE (Fig. 2). The results of amount and location of PVST in those 9 cases are shown in Table 2. All nine of these patients showed liver cirrhosis. According to the criteria of Tsamalaidze et al., thrombosis was classified as Type 1 in 2 cases, Type 2 in 1 case, Type 3 in 6 cases, and Type 4 in no cases. All 9 cases were asymptomatic, with 5 cases treated using additional anticoagulation. Two of those 5 cases received 2500 units of intravenous danaparoid sodium for 7 days, after which they were switched to warfarin. The remaining 3 cases received warfarin. Four cases did not receive any additional treatment for PVST.

**Table 2:** Amount and location of thrombus in the 9 cases with appearance or growth of PVST after PSE

	Age	Sex	PVST volume before PSE (ml)	PVST volume after PSE (ml)	Thrombus location after PSE
Case 1	58	M	0	1.14	SPV
Case 2	52	M	1.26	3.80	IHPV
Case 8	42	M	0	16.72	IHPV, PV
Case 17	72	F	1.07	3.91	IHPV, PV, SPV
Case 21	71	M	5.44	12.40	PV, SMV
Case 42	64	M	2.45	6.33	PV
Case 55	60	F	0	9.56	SPV
Case 59	71	F	0.92	3.27	IHPV, PV, SMV
Case 64	67	M	2.21	4.50	PV, SMV

*PVST* portal venous system thrombosis; *SPV* splenic vein; *IHPV* intrahepatic portal vein; *PV* portal vein; *SMV* superior mesenteric vein

### Factors Associated with Appearance or Growth of PVST After PSE

**Fig. 3:**
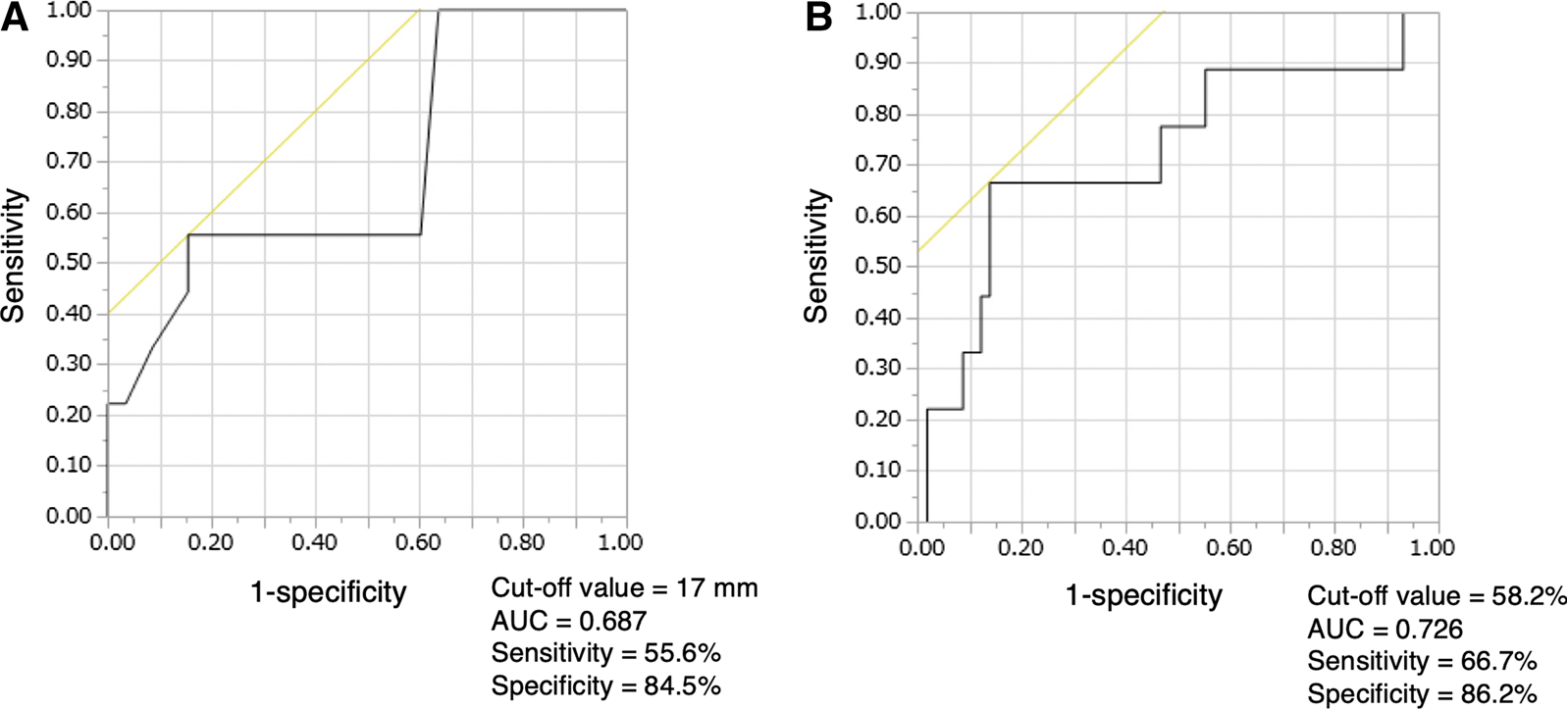
Receiver–operator characteristic curve prediction of the appearance or development of portal vein thrombosis after PSE. **A** Maximum diameter of the splenic vein before PSE. **B** Percentage of infarcted spleen

Uni- and multivariate analyses were performed for each covariate, divided according to the presence or absence of the appearance or development of PVST. The results are shown in Table 3. In univariate analyses, significant differences were identified for three factors before treatment: large maximum diameter of the splenic vein (*p* = 0.0076); large infarcted splenic volume (*p* = 0.0216); and high percentage of infarcted spleen (*p* = 0.0171). In multivariate analysis, significant differences were identified in two factors: large maximum diameter of the splenic vein (*p* = 0.0409, OR = 1.535, 95% CI = 1.018–2.315); and high percentage of infarcted spleen (*p* = 0.0230, OR = 1.136, 95% CI = 1.018–1.267). According to the ROC analysis, cutoff values to distinguish the appearance or development of PVST were maximum diameter of the splenic vein, 17 mm (*p* = 0.0306, AUC = 0.69, sensitivity = 55.6%, specificity = 84.5%); and percentage of infarcted spleen, 58.2% (*p* = 0.016, AUC = 0.73, sensitivity = 66.7%, specificity = 86.2%) (Fig. 3).

**Table 3:** Uni- and multivariate analysis of risk factors for appearance or development of PVST after PSE in the 67 patients

Variables	No. of patients	No. without appearance or growth of PVST after PSE	No. with appearance or growth of PVST after PSE	Univariate analysis		Multivariate analysis	
				*p* value	OR (95% CI)	*p* value	OR (95% CI)
Age				0.3056			
Sex				1.0000	0.9 (0.237–3.579)		
Male	46	40	6				
Female	21	18	3				
Platelet count				0.0507		0.2663	0.752 (0.455–1.243)
Prothrombin time				0.5911			
Total bilirubin				0.5518			
Albumin				0.4868			
ALT				0.4203			
Hepatic encephalopathy				1.0000	0.512 (0.026–10.041)		
Presence	5	5	0				
Absence	62	53	9				
ALBI score				0.5161		0.0725	47.820 (0.702–3235.2)
Hepatocellular carcinoma				0.4465	2.292 (0.158–16.82)		
Presence	4	3	1				
Absence	63	55	8				
Occlusion or thrombus of the portal venous system before PSE				0.0601	4.444 (1.15–17.22)	0.0539	10.665 (0.962–118.29)
Presence	24	18	6				
Absence	43	40	3				
Pre-PSE maximum diameter of the splenic vein				0.0076		0.0409	1.535 (1.018–2.315)
Pre-PSE total splenic volume				0.1528			
Infarcted splenic volume				0.0216		0.0886	0.995 (0.989–1.001)
Infarcted splenic percentage				0.0171		0.0230	1.136 (1.018–1.267)
Steroid used for pain management				0.4661	2.3 (0.487–11.6)		
Used	25	23	2				
Not used	42	35	7				
Heparin used after PSE				0.1249	0.275 (0.057–1.208)	0.1336	10.746 (0.483–239.20)
Used	10	7	3				
Not used	57	51	6				

*PVST* portal venous system thrombosis; *PSE* partial splenic artery embolization; *ALT* alanine aminotransferase; *ALBI* albumin-bilirubin;

### Maximum Diameter of the Splenic Vein Before and After PSE

The paired *t* test was performed on the maximum splenic vein diameter before and after PSE. Nine cases with appearance or growth of PVST (*p* = 0.0027), 58 cases without appearance or growth of PVST (*p* < 0.0001), and a total of 67 cases (*p* < 0.0001) showed significant decrease in maximum diameter of the splenic vein after PSE.

## Discussion

The possibility of PVST appearing as a complication after PSE is well known. Since PVST is associated with an increased risk of mortality [9, 10], assessing the risk of this complication before PSE is important. In this study, both uni- and multivariate analyses showed an association between maximum diameter of the splenic vein and the appearance and development of PVST. To the best of our knowledge, no previous reports have identified an association between diameter of the splenic vein and PVST after PSE, making this the first report to do so. In this study, the cutoff value for maximum diameter of the splenic vein before PSE calculated by ROC analysis as a risk factor for portal vein thrombus appearance or growth was 17 mm. Values of AUC (0.687) and sensitivity (55.6%) in this study were significant, but not very high. However, measurement of the maximum splenic vein diameter is easy and may offer a useful indicator to determine the extent of embolization before PSE. Further investigation is still required to enhance the validity of the results with more data.

In splenectomy performed for purposes similar to PSE, several investigators have reported associations with preoperative diameter of the splenic vein as a risk factor for portal and splenic venous thrombus [17–20]. Danno et al. reported an association between decreased diameter of the splenic vein and a decrease in portal and splenic venous blood flows [17]. This study showed a significant decrease in maximum splenic vein diameter on CT after PSE compared with before PSE, which may be associated with reduced splenic and portal venous flows. Kinjo et al. also reported that postoperative portal vein blood flow on ultrasonography was significantly reduced in patients with portal vein thrombus compared with patients without such thrombus, and the ratio of changes in portal vein blood flow before and after splenectomy correlated with preoperative splenic venous diameter as assessed by CT [18]. The exact mechanisms underlying the appearance or growth of PVST after PSE and splenectomy remain unclear. Kuroki et al. speculated that hypovolemia, congestion, and stagnation of blood flow in the splenic vein may be associated with the appearance or increase in portal vein thrombus after splenectomy [19]. We speculated that the larger the pre-treatment diameter of the splenic vein, the lower the blood flow in the portal venous system and the higher the risk of thrombus formation after PSE, as after splenectomy.

Several investigators have reported associations between splenic infarction percentages and splenic infarction volume and post-PSE complications. Zhu et al. reported that when 62 patients undergoing PSE were divided into three groups with percentages of infarcted spleen of < 50%, 50–70%, and > 70% as measured by CT, serious complications including PVST were significantly higher in the > 70% group [4]. Hayashi et al. reported that among 71 patients who underwent PSE, risk factors for complications differed significantly between uni- and multivariate analyses for splenic infarction volume ≥ 540 mL. However, PVST was not included as a complication in this study [1]. Cai et al. examined risk factors for eight patients with serious complications among 52 patients who underwent PSE. The study included 3 cases of PVST out of 8 complications. They reported significant differences in splenic infarct volume in multivariate analysis, and cutoff values were 513.1 mL in ROC analysis [2]. These reports and our results suggest that a large splenic infarct volume or a high percentage of infarcted spleen is associated with an increased risk not only for all complications of PSE but also for the appearance of PVST only.

Several investigators have reported the safety and efficacy of thrombolytic therapy for PVST by anticoagulant and antithrombin III [20–23]. However, use of thrombolytic therapy for PVST in patients with liver cirrhosis is currently controversial. The American Association for the Study of Liver Diseases guideline for the management of PVST in liver cirrhosis was published in 2009 and recommended anticoagulation for acute PVST in patients with cirrhosis be given on a case-by-case basis, depending on prothrombin status, symptoms, and thrombus progression to SMV. Ten of the 67 patients in this study (14.9%) received intravenous heparin after PSE for the purpose of preventing PVST. However, no significant differences were seen in the appearance or growth of PVST with intravenous heparin after PSE. Detection of PVST may thus be more important than prophylactic treatment.

Some limitations must be considered when interpreting the findings from this study. First, this study was used a retrospective design. The duration of CECT before and after treatment was thus not constant. Conducting a prospective study to evaluate both pre- and post-treatment CT with the same duration is thus desirable. In addition, embolization materials were also inconsistent. Second, differences in splenic venous flow rate by ultrasonography before and after PSE were not evaluated. We speculated an association between PSE-induced reduction in splenic venous flow and the appearance and growth of PVST. However, the evaluation of changes in splenic venous flow rate is necessary to prove this association. Third, few patients were treated with anticoagulation therapy. Only 10 patients underwent intravenous heparin anticoagulation after PSE, because intravenous heparin has been administered after PSE in patients other than those with emergency splenic hemorrhage since September 2015.

## Conclusion

This study suggests that a large maximum diameter of the splenic vein (≥ 17 mm) on CT before PSE and a high percentage of infarcted spleen (≥ 58.2%) are associated with the risk of PVST appearance and growth after PSE. When the maximum diameter of the splenic vein is large (≥ 17 mm) before PSE, earlier testing may be recommended to detect the development of thrombus.
